# MdMAPK6-mediated phosphorylation of MdWRKY9 regulates apple fruit ripening through interaction with MdERF5L

**DOI:** 10.1093/hr/uhaf200

**Published:** 2025-07-31

**Authors:** Hui Wang, Shu-Hui Zhang, Yu-Chen Feng, Jia-Hao Zhao, Tong Wang, Wen-Jun Liu, Xue-Sen Chen, Nan Wang

**Affiliations:** State Key Laboratory of Crop Stress Biology for Arid Areas, College of Horticulture, Northwest A&F University, Yangling, Shaanxi 712100, China; State Key Laboratory of Crop Stress Biology for Arid Areas, College of Horticulture, Northwest A&F University, Yangling, Shaanxi 712100, China; State Key Laboratory of Crop Stress Biology for Arid Areas, College of Horticulture, Northwest A&F University, Yangling, Shaanxi 712100, China; College of Horticultural Science and Engineering, Shandong Agricultural University, State Key Laboratory of Crop Biology, Taian, Shandong 271018, China; College of Horticultural Science and Engineering, Shandong Agricultural University, State Key Laboratory of Crop Biology, Taian, Shandong 271018, China; College of Horticultural Science and Engineering, Shandong Agricultural University, State Key Laboratory of Crop Biology, Taian, Shandong 271018, China; College of Horticultural Science and Engineering, Shandong Agricultural University, State Key Laboratory of Crop Biology, Taian, Shandong 271018, China; College of Horticultural Science and Engineering, Shandong Agricultural University, State Key Laboratory of Crop Biology, Taian, Shandong 271018, China

## Abstract

WRKY transcription factors are essential for mediating many developmental processes, such as fruit ripening, a highly controlled and intricate physiological phenomenon. In the current research, a novel transcription factor, MdWRKY9, was identified and categorized, which significantly promotes apple fruit ripening. Its role was validated through a combination of transient injection and stable overexpression-transformed tomato experiments. Notably, MdWRKY9 interacts with the fruit ripening suppressor MdERF5L at protein and DNA levels. This interaction counteracts MdERF5L-mediated MdACS1 repression. MdACS1 is an important ethylene biosynthesis enzyme; the mentioned process eventually allows fruit ripening. Furthermore, phosphorylation, a post-translational modification, regulates maturation and ethylene synthesis. Through liquid chromatography–tandem mass spectrometry, we identified phosphorylation sites within the MdWRKY9-GFP protein. MdMAPK6–MdWRKY9 interaction and MdMAPK6-mediated phosphorylation of MdWRKY9 were confirmed through protein–protein interaction assays, such as bimolecular fluorescence complementation (BIFC), yeast two-hybrid (Y2H), protein phosphorylation, luciferase complementation imaging (LCI), and pull-down assays. Specifically, MdMAPK6 phosphorylates MdWRKY9 at Tyr394 site, enhancing the stability and activity of MdWRKY9 and thus modulating its regulatory role in fruit maturation. These findings provide new directions and insights into the intricate regulatory network governing the ripening of apples.

## Introduction

A crucial physiological process that takes place in the latter phases of fruit growth and hints at the end of plant reproduction is the ripening of fruits. Numerous physiological and biochemical alterations are involved, including sugar buildup, color changes, cell wall disintegration, and the release of aromatic chemicals [1, 2]. Precisely controlling the ripening stages is crucial to ensure timely harvesting and optimal storage conditions, making it essential to understand the processes by which ripening occurs. Several metabolic processes mediate the change from the unripe to the ripe stage of fruit growth, particularly those involving plant hormones, making it a tightly regulated and highly coordinated process [3]. Ripening in climacteric fruits is characterized by a sharp rise in the levels of ethylene. Ethylene biosynthesis primarily takes place in two stages: (i) ACC synthase (ACS) catalyzes 1-aminocyclopropane-1-carboxylic acid (ACC) synthesis from S-adenosylmethionine, and (ii) ACC oxidase (ACO) catalyzes ACC transformation into ethylene [4]. ACS is the essential catalyst in the production of ethylene since it is the rate-limiting process in the formation of ACC [5]. In a study, silencing *MdACS1* in apple fruits inhibited the respiratory climacteric phase, preventing normal ripening, which underscores the critical role of ACS in controlling ripening [6]. Regulating ethylene biosynthesis, particularly through the modulation of ACS activity, is the key to controlling climacteric fruit ripening [1, 7–9]. By directly influencing the expression of structural genes that are downstream in the ethylene signaling pathway, the transcription factors termed ethylene response factors (ERFs) operate as the main mediators in this mechanism. ERFs are also involved in ethylene biosynthesis, regulating ethylene levels released during fruit ripening [1, 10–13]. In bananas (*Musa acuminata*), MaERF9 binds to the promoter of *MaACS1* to activate it, whereas MaERF11 represses both *MaACS1* and *MaACO1* via binding to regulate the maturation of fruits [10]. In apples, MdERF2 can bind to the promoter of *MdACS1* to enhance its expression level, whereas MdERF3, upon binding to its promoter, inhibits the expression of *MdACS1* [1]. Although there is ample indication that ERFs play a crucial role in fruit ripening, their functional characterization is still lacking.

One of the biggest groups of transcription regulators found in terrestrial plants is represented by WRKY transcription factors. WRKY transcription factors, which are distinguished by their highly conserved WRKY domains, are essential for plant development, growth, and responses to both abiotic and biotic stressors [14–17]. There is growing evidence that WRKYs regulate the ripening of fruits [18–20]. *FvWRKY48*, *FvWRKY46*, and *FvWRKY4* exhibited high expression in strawberries during the development and ripening of fruits. *FvWRKY48* combines with the *FvPLA* promoter through the W-box element to promote fruit ripening and softening [18, 19]. Similarly, *PuWRKY31* has been shown to speed up fruit ripening in pears by binding to and triggering the promoters of *PuACS1a* and *PuACO1* in response to sucrose, hence increasing ethylene production [20]. Earlier, we recognized a novel WRKY family member in apple fruits—MdWRKY9 (NM_001294127); its expression was associated with sugar accumulation, a key indicator of fruit ripening [21]. Preliminary findings have confirmed that MdWRKY9 acts as a transcriptional activator of *MdSWEET9b*, mediating ABA’s role in fruit sugar accumulation through interactions with MdbZIP23 and MdbZIP46. Furthermore, while MdWRKY9 expression progressively increases during fruit development, its role in fruit ripening remains unexplored [21]. In this study, through transient injection, stable overexpression-converted tomato, and several molecular investigations, we elucidated the molecular mechanism by which MdWRKY9 promotes apple fruit ripening. This work provides new insights into WRKY-mediated regulation of fruit ripening.

In addition to the transcriptional-level regulation, post-translational modifications such as phosphorylation can also affect fruit ripening. Mitogen-activated protein kinase (MAPK) protein’s role in phosphokinase signaling in this process is well documented [22–25]. In tomato, SlMAPK4 regulates the transition from binucleate to trinucleate microspores during pollen development and affects parthenocarpic fruit formation [26]. In strawberry, MAPK6 phosphorylates FvRIF at Thr-310, modulating fruit ripening [27]. In banana, MaMPK6-3/11-4 phosphorylates the transcription factor MabZIP21 to regulate ripening [28], and MaMAPK14 phosphorylates MaMYB4 negatively to regulate fruit ripening [29]. The WRKY transcription factors are downstream targets of the MAPK cascades, where MAPK phosphorylates and modifies WRKY to enhance its DNA W-box-binding ability [30]. For example, AtMAPK4 phosphorylates and activates AtWRKY52 and AtWRKY33, amplifying their regulatory effects [31]. Tomato SlMAPK4 can phosphorylate SlWRKY6 to mediate its overvulcanization modification, thereby inhibiting fruit ripening [32]. This study reveals that apple MdMAPK6 interacts with and phosphorylates MdWRKY9 through a yeast double-impurity sieve library and a series of molecular biological methods, thereby enhancing its stability and functional activity. Collectively, these findings establish a novel molecular framework for precision-targeted regulation of apple fruit ripening, while advancing mechanistic insights into the multilayered regulatory network orchestrating fruit maturation.

## Results

### *MdWRKY9* stimulates ripening in apples and tomatoes

**Figure 1:**
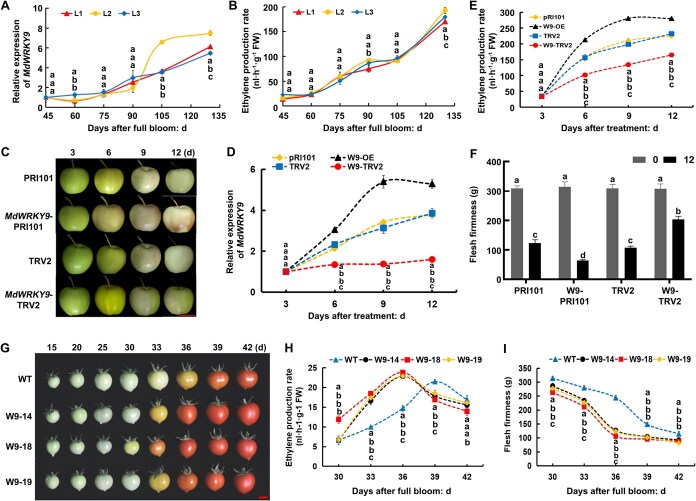
*MdWRKY9* promotes ripening in both tomatoes and apples. (A, B) *MdWRKY9* expression level (A) as well as ethylene release (B) during development of apples. L1, L2, and L3 represent different superior lines of apple hybrid offspring. (C–F) Phenotype (C), *MdWRKY9* expression level (D), ethylene release (E), and flesh firmness (F) of ‘Taishanzaoxia’ apple following transient injection of *MdWRKY9*. Bar = 4 cm. Phenotype (G), ethylene release (H), and flesh firmness (I) of tomato lines stably transformed with *MdWRKY9*. Bar = 1 cm. W9 is the abbreviation of *MdWRKY9*. The SD of three separate biological replicates is shown by error bars. Significant differences at *P* < 0.05 are indicated via various lowercase letters (Student’s *t-*test).

To investigate how MdWRKY9 helps in the ripening of fruits, three independent hybrids (‘*Gala*’ × ‘*CSR6R6*’) with the same maturation stage were randomly selected. *MdWRKY9* expression and ethylene release during fruit development were quantified. The results indicated that *MdWRKY9* expression increased progressively with fruit ripening, with significant increases noted during the advanced phases of fruit development. Ethylene release followed the same pattern, with a significant rise observed during the final development stages (Fig. 1A and B). These results indicated that *MdWRKY9* may play an important role in promoting the process of fruit ripening.

Subsequently, we established the *MdWRKY9* overexpression and virus-mediated gene silencing vectors, and inserted into ‘Taishanzaoxia’ apples through *Agrobacterium tumefaciens*-mediated vacuum infiltration to validate how MdWRKY9 affects fruit ripening. Fruits overexpressing *MdWRKY9* (*MdWRKY9*-PRI01) exhibited yellowing by Day 6 of infiltration, with local decay evident by Day 12 (Fig. 1C). Quantitative reverse transcription polymerase chain reaction (RT-qPCR) and semiquantitative analysis demonstrated significantly elevated *MdWRKY9* expression levels in fruits exhibiting gene overexpression compared with control (PRI101) fruits, with the expressions peaking on Day 9. This peak coincided with the maximum ethylene release, which was also significantly higher than that in control fruits. Furthermore, overexpression of *MdWRKY*9 significantly upregulated the ethylene biosynthesis genes *MdACS1* and *MdACO1*, and fruit firmness was markedly reduced compared to controls (Fig. 1D–F, Fig. S1). Conversely, fruits with silenced *MdWRKY9* (*MdWRKY9*-TRV2) showed delayed softening, reduced ethylene release, and downregulated the expression of *MdWRKY9*, *MdACS1*, and *MdACO1* (Fig. 1C–F, Fig. S1). To validate these findings, we analyzed the influence of *MdWRKY9* expression on transgenic tomato lines. In three independent *MdWRKY9* overexpression tomato lines, fruit ripening was accelerated by 3–8 days (Fig. S2). These lines exhibited increased ethylene release rates, an earlier ethylene release peak, and reduced fruit firmness (Fig. 1G–I). Collectively, these results confirmed that *MdWRKY9* promotes ripening in both tomatoes and apples.

### MdWRKY9 interacts with MdERF5L to regulate ripening in apples

**Figure 2:**
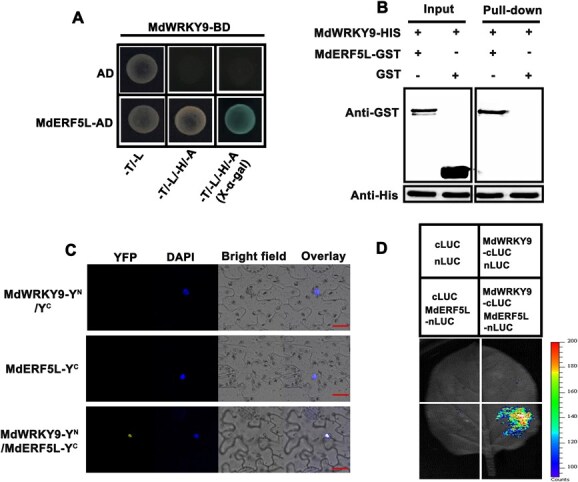
MdWRKY9 interacts with MdERF5L to regulate ripening in apples. (A) MdWRKY9 and MdERF5L interaction is demonstrated by Y2H assays using the empty pGADT7 vector (AD) as a negative control. (B) Pull-down assays verified that MdWRKY9 and MdERF5L interacted. ‘+’ and ‘−’ denote the presence and lack of the designated protein, respectively. (C) BIFC assays confirmed the MdWRKY9–MdERF5L interaction. (D) LCI tests showing interaction between MdERF5L and MdWRKY9.

To gain further insights into the regulatory mechanism of MdWRKY9, we employed MdWRKY9-BD as the bait protein in a yeast two-hybrid (Y2H) screen. The findings showed that MdWRKY9 links with MdERF5L, a crucial element of the ethylene signaling cascade (Table S1). Specific Y2H assay confirmed this interaction: MdWRKY9-BD and MdERF5L-AD cotransformed yeast cells grew normally and turned blue on selective media deficient in leucine, tryptophan, adenine, and histidine (−L/−T/−A/−H; Fig. 2A). Subsequently, using a pull-down assay, the relationship between MdERF5L and MdWRKY9 was further confirmed, where MdERF5L-GST successfully pulled down MdWRKY9-His, while the GST control could not (Fig. 2B). Additionally, when MdWRKY9-YFP^N^ and MdERF5L-YFP^C^ were coexpressed, the results of bimolecular fluorescence complementation (BiFC) showed a robust yellow fluorescent protein (YFP) signal (Fig. 2C). Finally, luciferase complementation imaging (LCI) demonstrated that co-injection of MdWRKY9-LUC^C^ and MdERF5L-LUC^N^ into tobacco leaves produced a significantly stronger fluorescence signal than the controls groups (Fig. 2D). All of these findings provided evidence that MdWRKY9 and MdERF5L interact both *in vivo* and *in vitro*.

### Fruit ripening is inhibited when MdERF5L attaches to the *MdACS1* promoter and suppresses its activity

**Figure 3:**
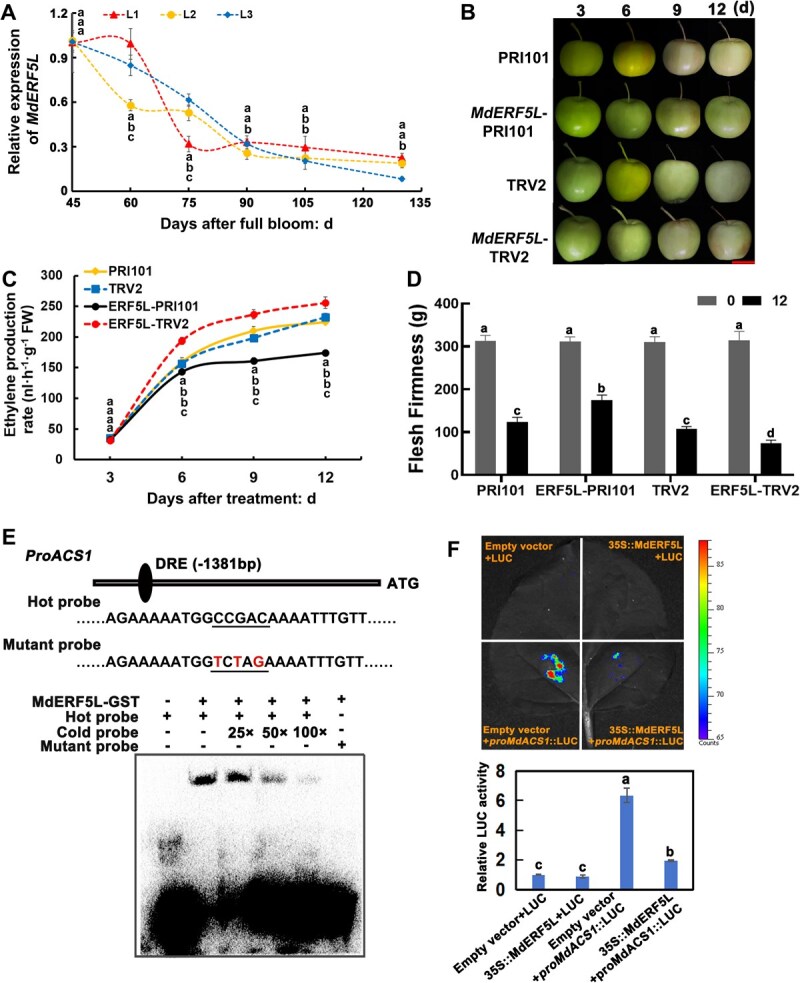
MdERF5L inhibits fruit ripening by binding to the *MdACS1* promoter and blocking its activity. (A) The *MdERF5L* expression level in apple fruits during development. L1, L2, and L3 represent different superior lines of apple hybrid offspring. (B–D) Phenotype (B), ethylene release (C), and flesh firmness (D) of ‘Taishanzaoxia’ apple after the transient injection of *MdERF5L*. Bar = 4 cm. (E, F) EMSA (E) and luciferase reporter assay (F) showing how MdERF5L binds to the *MdACS1* promoter both *in vivo* and *in vitro*. ‘−’ and ‘+’ denote the lack and presence of the protein or probe, respectively. The SD of three separate biological replicates is shown by error bars. Significant differences at *P* < 0.05 are indicated via various lowercase letters (Student’s *t-*test).

During fruit ripening, the ERF transcription factor family is essential for controlling ethylene levels. To investigate MdERF5L’s role in this process, we first performed RT-qPCR on the developing apple fruits. Our results revealed a progressive downregulation of *MdERF5L* expression as the fruits ripened, which led us to hypothesize that MdERF5L might act as a negative regulator of ripening (Fig. 3A). To validate this hypothesis, we conducted a transient injection experiment. *MdERF5L* overexpression in apple fruits delayed ripening, as indicated by reduced ethylene release and delayed softening compared with control fruits (PRI101). Conversely, *MdERF5L* silencing accelerated ripening, leading to increased ethylene release and accelerating softening compared with those in control fruits (TRV2) (Fig. 3B–D; Fig. S3). Moreover, exogenous ethephon could partially alleviate the inhibitory effect of *MdERF5L* overexpression on fruit ripening (Fig. S4). Together, these results confirmed that *MdERF5L* inhibits ethylene synthesis and negatively regulates fruit ripening.

Since MdACS1 plays a positive regulatory function in apple ripening and ethylene production [1], and *cis*-acting element analysis of its promoter revealed the presence of ERF transcription factor-binding sites, we investigated whether MdERF5L directly inhibits the activity of *MdACS1*. Electrophoretic mobility shift assays (EMSA) indicated that MdERF5L binds to the *MdACS1* promoter. The addition of a cold probe and mutations in the *MdACS1* promoter sequence disrupted this binding (Fig. 3E). A luciferase reporter assay (LUC) also confirmed this interaction. The presence of MdERF5L significantly reduced *MdACS1* promoter activity (Fig. 3F). According to these statements, MdERF5 binds to the promoter of *MdACS1*, suppressing its expression and thus inhibiting apple fruit ripening.

### MdWRKY9 modulates MdERF5L-mediated regulation of *MdACS1* expression

**Figure 4:**
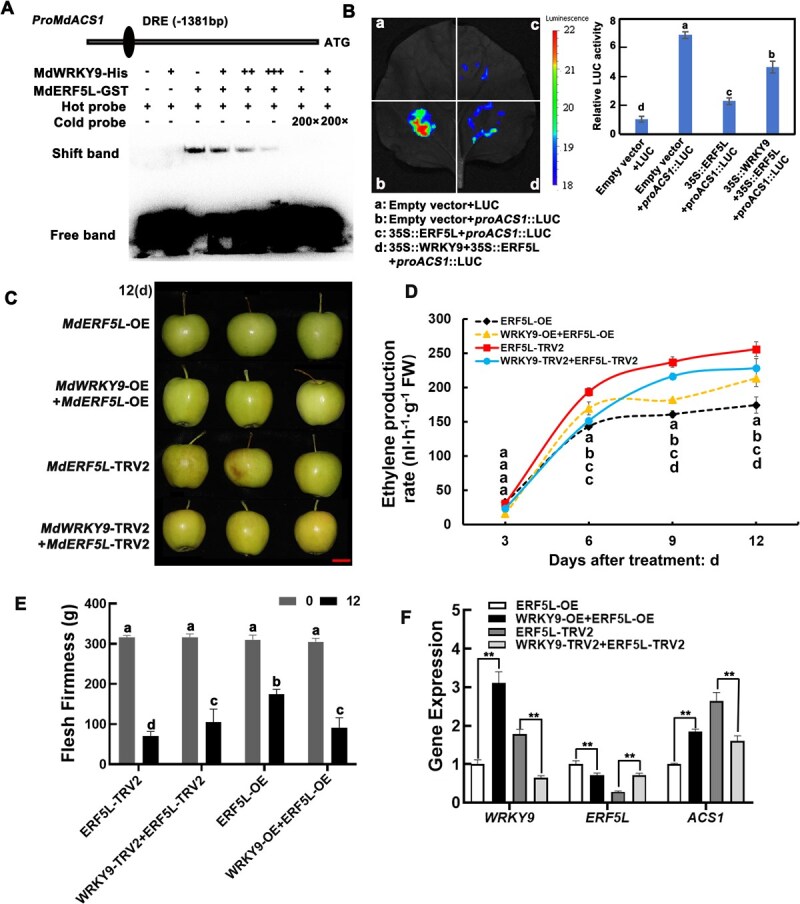
MdWRKY9 interacts with MdERF5L, weakening the regulatory effect of MdERF5L on *MdACS1* and promoting fruit ripening. (A, B) EMSA (A) and luciferase assay (B) demonstrate that MdWRKY9’s association with MdERF5L prevents MdERF5L’s regulatory effect on *MdACS1*. ‘−’ and ‘+’ indicate the lack and presence of the mentioned protein or probe, respectively. (C–F) Phenotype (C), ethylene release (D), flesh firmness (E), and gene expression level (F) of ‘Taishanzaoxia’ apple after the transient injection of *MdERF5L* and *MdWRKY9*. Bar = 4 cm. Error bars represent the SD of three independent biological replicates. Different lowercase letters indicate significant differences at *P <* 0.05 (Student’s *t*-test). SPSS, version 22, was used to analyze the correlation between the data (^**^P < 0.01, ^*^P < 0.05) (Student’s *t-*test).

To discover how the link between MdWRKY9 with MdERF5L influences fruit ripening, we conducted the EMSA, which revealed the binding dynamics of these proteins. Increase in the concentrations of MdWRKY9 gradually weakened MdERF5L’s ability to bind to the *MdACS1* promoter (Fig. 4A). This finding was corroborated *in vivo* through the LUC assay. While MdERF5L inhibited *MdACS1* promoter activity, the addition of MdWRKY9 diminished this inhibitory effect (Fig. 4B). These results indicated that MdWRKY9 interferes with MdERF5L’s binding to the *MdACS1* promoter, thereby reducing MdERF5L-mediated suppression of *MdACS1* expression.

In addition, the effect of the MdWRKY9–MdERF5L interaction on fruit ripening was investigated by instantaneously co-injecting apples. Simultaneous *MdWRKY9* and *MdERF5L* overexpression significantly promoted fruit ripening. Compared with the overexpression of *MdERF5L* alone, simultaneous overexpression of *MdWRKY9* led to increased ethylene release, decreased fruit hardness, and increased *MdACS1* expression levels (Fig. 4C–F). In *MdERF5L*-TRV2-silenced fruits, additional silencing of *MdWRKY9* significantly inhibited *MdERF5L* silencing-mediated over-ripening effects. Compared with fruits silenced by *MdERF5L*-TRV2 alone, *MdWRKY9* silencing inhibited ethylene release, delayed fruit hardness reduction, and significantly inhibited *MdACS1* (Fig. 4C–F), which confirmed that *MdWRKY9* promotes fruit ripening by modulating *MdERF5L*’s regulatory effect on *MdACS1*. Collectively, these results indicated that MdWRKY9 interacts with MdERF5L, weakening its *MdACS1* suppression ability and thereby promoting fruit ripening.

### MdWRKY9 directly downregulates *MdERF5L* expression by binding to its promoter

**Figure 5:**
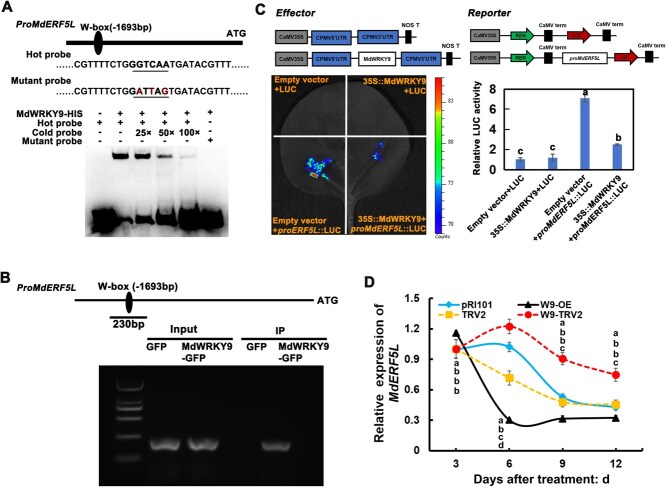
MdWRKY9 transcriptionally suppresses *MdERF5L* expression through direct binding to its promoter region, thereby promoting fruit ripening. (A) Assays for electrophoretic mobility shift demonstrate that MdWRKY9 binds to the *MdERF5L* promoter. ‘−’ and ‘+’ indicate the lack and presence of the mentioned protein or probe, respectively. (B) DNA fragments enriched in each ChIP-PCR test are used as the biological replication, which shows that MdWRKY9 binds to the *MdERF5L* promoter *in vivo*. (C) Results of the luciferase assay demonstrate that MdWRKY9 binds to the *MdERF5L* promoter *in vivo*. (D) *MdWRKY9* transgenic apples express *MdERF5L*. The SD of three separate biological replicates is shown by error bars. Significant differences at *P* < 0.05 are indicated via various lowercase letters (Student’s *t-*test).

Based on the data obtained through RT-qPCR, overexpression of *MdWRKY9* alters the expression of *MdERF5L* (Fig. 4F). This prompted us to speculate that MdWRKY9 might directly regulate *MdERF5L* expression. Analysis of the MdERF5L promoter revealed the presence of a W-box binding site, which is a target for WRKY transcription factors. We designed a specific probe, and performed an EMSA assay, confirming that MdWRKY9 indeed specifically binds to the *MdERF5L* promoter (Fig. 5A). Subsequently, the chromatin immunoprecipitation-PCR (ChIP-PCR) experiment further validated this interaction *in vivo* (Fig. 5B). The LUC assay further demonstrated that MdWRKY9 binding suppresses *MdERF5L* promoter activity (Fig. 5C). Additionally, the expression of *MdERF5L* in *MdWRKY9*-overexpressing fruits was measured. Consistently, *MdWRKY9* overexpression inhibited *MdERF5L* expression, whereas *MdERF5L* expression significantly increased as a result of *MdWRKY9* silencing (Fig. 5D). These results offer strong evidence supporting that MdWRKY9 directly binds the *MdERF5L* promoter to inhibit its expression, eventually promoting apple fruit ripening.

### MdMAPK6 interacts with MdWRKY9 both *in vivo* and *in vitro*

**Figure 6:**
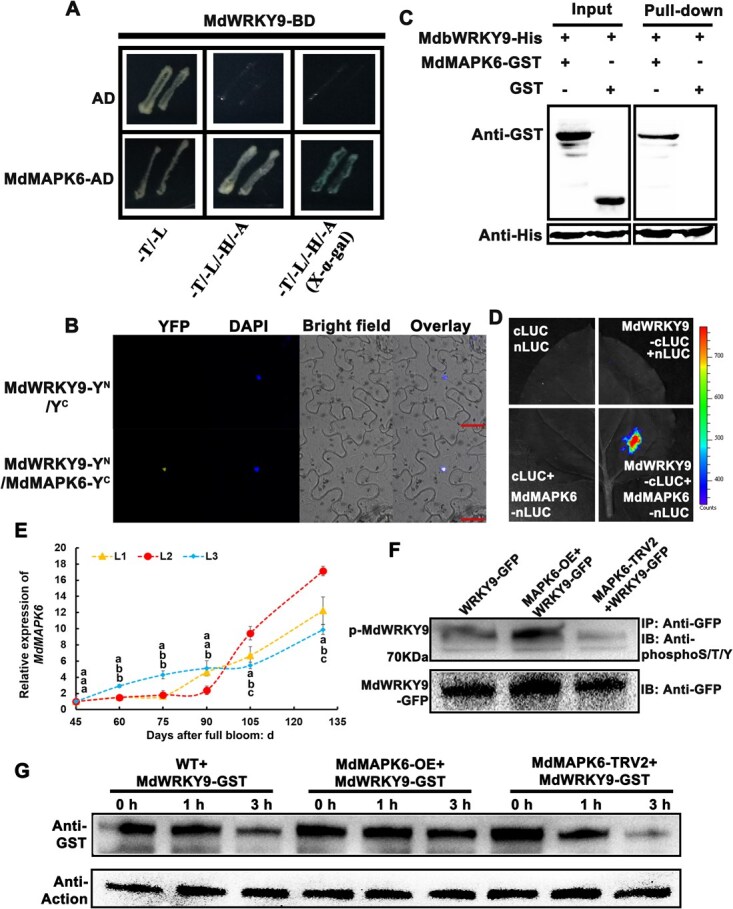
MdMAPK6 binds to MdWRKY9 both *in vivo* and *in vitro.* (A) Two-hybrid tests using yeast show how MdMAPK6 and MdWRKY9 interact. The negative control is the empty pGADT7 vector (AD). (B) Assays using bimolecular fluorescence complementation show how MdMAPK6 and MdWRKY9 interact. (C) Pull-down tests show how MdMAPK6 and MdWRKY9 interact; ‘−’ and ‘+’ indicate the lack and presence of the mentioned protein, respectively. (D) LCI confirmed that MdMAPK6 interacts with MdWRKY9. (E) The *MdMAPK6* expression level in apple fruits during development. L1, L2, and L3 represent different superior lines of apple hybrid offspring. (F) MdMAPK6-mediated phosphorylation of MdWRKY9 *in vivo*. Proteins were separated using SDS-PAGE for immunoblot analysis after being immunoprecipitated from transgenic apple calli (35S: GFP-MdWRKY9, 35S: GFP-MdWRKY9 + TRV2-MdMAPK6, and 35S: GFP-MdWRKY9 + 35S:MdMAPK6) using an anti-GFP antibody. An anti-phosphoS/T/Y antibody was used to detect MdWRKY9 phosphorylation. (G) The protein extract of apple calli with *MdMAPK6* overexpression (MdMAPK6-OE) demonstrated a reduced rate of MdWRKY9 degradation and a higher rate of *MdMAPK6* silencing (MdMAPK6-TRV2) relative to that in the WT calli in an *in vitro* cell-free degradation test.

Phosphorylation plays a pivotal role in modulating ethylene synthesis and maturation. Given that WRKY transcription factors are often targets of phosphorylation, we investigated whether MdWRKY9 undergoes this post-translational modification. Purified MdWRKY9-GFP protein from transgenic calli with stable *MdWRKY9* overexpression was analyzed using liquid chromatography–tandem mass spectrometry (LC–MS/MS). Phosphorylated peptides confirmed that MdWRKY9 undergoes phosphorylation (Table S2). Protein–protein interaction studies using Y2H revealed an interaction between MdMAPK6 and MdWRKY9 (Fig. 6A). This interaction was subsequently validated through BiFC, pull-down, and LCI tests, which established that the protein–protein interaction between MdMAPK6 and MdWRKY9 (Fig. 6B–D). Meanwhile, according to the results of RT-qPCR, *MdMAPK6* expression rose as fruit ripening advanced (Fig. 6E). The phosphorylation level of MdWRKY9 rose with *MdMAPK6* overexpression but fell with *MdMAPK6* silencing, according to incubation with anti-phosphoS/T/Y antibody after *MdMAPK6*’s transient overexpression as well as silencing in *MdWRKY9*-GFP transgenic calli (Fig. 6F). We then extracted the crude protein of the calli with *MdMAPK6* overexpression and silencing. Taking the wild-type (WT) callus as the control, we found that after incubation with MdWRKY9-GST for 0, 1, and 3 h, the degradation of MdWRKY9 accelerated in the crude protein with silenced *MdMAPK6* but slowed in those overexpressing *MdMAPK6* (Fig. 6G). Taken together, these results indicated that MdMAPK6 interacted with and phosphorylated MdWRKY9, and the phosphorylation of MdWRKY9 conferred it with greater stability.

### MdMAPK6 phosphorylates MdWRKY9 at Tyr394, stabilizing its protein structure and amplifying its effect on fruit ripening

**Figure 7:**
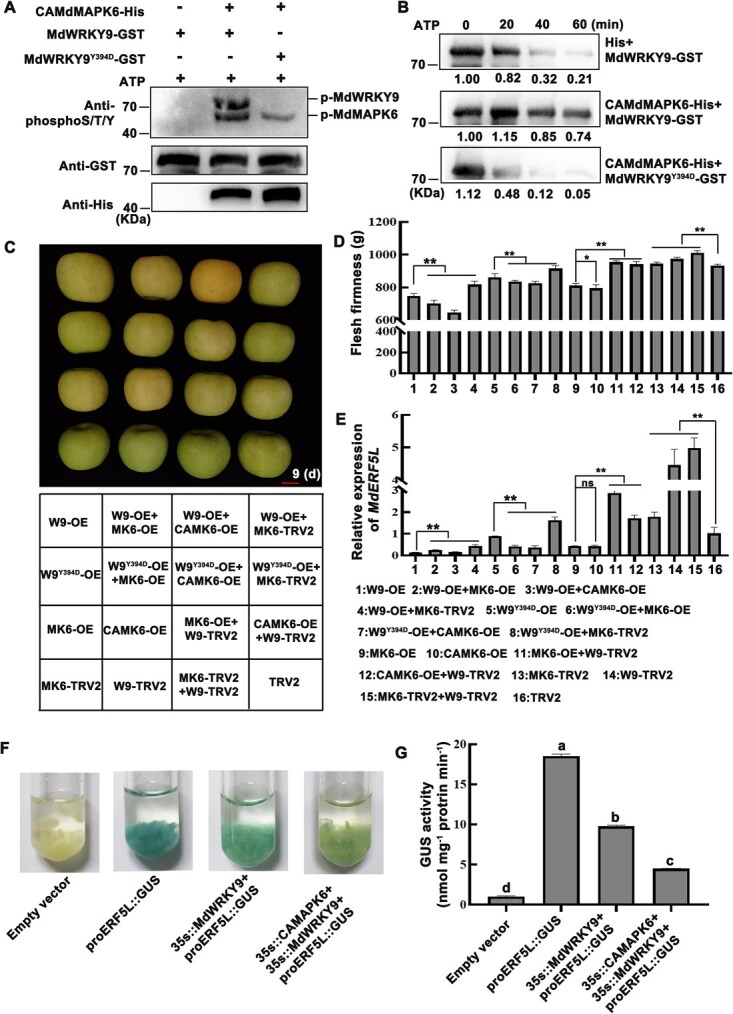
MdMAPK6 phosphorylates MdWRKY9 at Tyr394 and stabilizes MdWRKY9 structure, thereby promoting fruit ripening. (A) MdMAPK6-induced *in vitro* MdWRKY9 phosphorylation at the Tyr394 site. Following production in BL21 (DE3), recombinant MdWRKY9-GST, MdWRKY9^Y394D^-GST (a mutant lacking the phosphorylation site), and CAMdMAPK6-His were extracted and utilized for phosphorylation tests. ‘−’ and ‘+’ indicate the lack and presence of the mentioned protein, respectively. (B) MdWRKY9-GST degradation is inhibited by CAMdMAPK6-His, according to an *in vitro* cell-free degradation test. His protein was used as a control, and ATP helped in incubating equally purified CAMdMAPK6-His with the recombinant MdWRKY9-GST protein or MdWRKY9^Y394D^-GST protein. An anti-GST antibody was used to determine the extent of protein breakdown following incubation for 0, 20, 40, and 60 min. The number underneath the band indicates the relative abundance measured using density scan, with the protein abundance of the CAMdMAPK6-His + MdWRKY9-GST mixture incubated for 0 min set to ‘1.00’. (C–E) Phenotype (C), flesh firmness (D), and *MdERF5L* gene expression level (E) in instantaneously injected ‘Ruixue’ apples. W9 represents WRKY9, MK6 represents MdMAPK6, and CAMK6 represents CAMdMAPK6. (F–G) The GUS staining phenotype (F) and GUS activity (G) analysis in *ProERF5L*::GUS calli transiently expressing *MdWRKY9* and *MdMAPK6*. Error bars represent the SD of three independent biological replicates. Different lowercase letters indicate significant differences at *P <* 0.05 (Student’s *t-*test). SPSS, version 22, was used to analyze the correlation between the data (^**^P < 0.01, ^*^P < 0.05) (Student’s *t-*test).

IP/MS techniques identified Tyr394 as a phosphorylation site on MdWRKY9 (Fig. S5, Table S2). To verify whether MdMAPK6 phosphorylates MdWRKY9 at Tyr394, we performed *in vitro* phosphorylation assays in kinase buffer by using the self-phosphorylating active CAMdMPK6-His protein and the MdWRKY9^Y394D^-GST protein with Tyr394-to-Asp substitution as a point mutation. Following incubation of the proteins with the anti-phosphoS/T/Y antibody, we observed that CAMdMAPK6-His phosphorylated MdWRKY9-GST but failed to phosphorylate the mutant MdWRKY9 protein (MdWRKY9^Y394D^-GST) (Fig. 7A). Using empty His protein as a reference, we incubated purified CAMdMAPK6-His with MdWRKY9^Y394D^-GST and MdWRKY9-GST *in vitro* to further ascertain the impact of phosphorylation on the stability of MdWRKY9. The degradation of MdWRKY9 by CAMdMAPK6-His slowed down over time but accelerated after Tyr394 mutation than the control group, indicating that MdMAPK6 enhanced the stability of MdWRKY9 and that Tyr394 plays a key role in this process (Fig. 7B).

To further verify the biological function of MdWRKY9 after phosphorylation, we vacuum-injected ‘Ruixue’ fruit with *MdMAPK6* and *MdWRKY9*. As shown in Fig. 7C–D, silencing of *MdMAPK6* and *MdWRKY9* alone or together significantly inhibited the reduction of ripening and firmness in fruits. Moreover, *MdWRKY9* promoted a greater drop in fruit ripening and firmness than *MdWRKY9^Y394D^*, which was enhanced by *MdMAPK6* overexpression and suppressed by *MdMAPK6* silencing, while *CAMAPK6* had a more significant effect on this process, indicating that MdMAPK6 could stabilize the activity of MdWRKY9, thereby promoting ripening of fruits. Subsequently, *MdERF5L* gene expression further confirmed this result. Silencing of *MdMAPK6* and *MdWRKY9* promoted the expression of mature suppressant *MdERF5L*, while overexpression inhibited its expression. Moreover, the inhibitory effect of *MdWRKY9* on *MdERF5L* was more obvious than that after *MdWRKY9^Y394D^* mutation. The overexpression of *MdMAPK6* promoted the inhibitory effect of MdWRKY9 on *MdERF5L*. In addition, the GUS reporting system also showed that MdWRKY9 inhibited *MdERF5L* promoter activity, and this inhibitory effect was amplified by the addition of MdMAPK6 (Fig. 7F and G; Fig. S5). In conclusion, MdMAPK6 phosphorylates MdWRKY9 at Tyr394, stabilizing MdWRKY9 structure and enhancing its positive effect on fruit ripening.

## Discussion

Environmental and genetic variables regulate the highly synchronized biochemical and physiological events that occur during the ripening of fruits [33–35]. WRKY transcription factors are the vital factors that control a variety of plant growth processes [18–20]. In crops, such as chili and strawberry, CaWRKY (nearly 60%) and FvWRKY48 exhibited upregulation during fruit ripening, indicating how WRKY transcription factors affect maturation as well as softening [18, 36]. Nevertheless, little is known about the methods by which WRKY transcription factors control fruit ripening. According to a study, FvWRKY48 accelerates the softening and ripening of strawberry fruit by binding to the W-box element in the *FvPLA* promoter to increase pectin lyase function [19]. In line with these inferences, the current work established a strong basis for future research into the mechanisms of action by which the WRKY family affects the ripening of fruits by confirming how MdWRKY9 mediates in apple ripening.

The WRKY family influences plant growth, development, and disease resistance by interacting with other proteins. For example, in response to powdery mildew, the disease-resistant R protein MLA in barley interacts with WRKY proteins (HvWRKY1 and HvWRKY2), thus relieving the inhibitory effect of these proteins on disease resistance [37]. VviWRKY03 interacts with VviMYB14 to co-activate *VviSTS29* promoter activity and regulate resveratrol biosynthesis in grapevines [38]. In this study, we found that MdWRKY9 interacts with MdERF5L, a transcription factor belonging to the ERF family. ERFs are commonly recognized elements of the ethylene signal transduction cascade that are essential for controlling the ripening of fruits [1, 10–13]. Ethylene, a critical phytohormone in respiratory climacteric fruits, is the most direct signal that regulates the ripening of fruits. It drives maturation via transcriptionally controlling key genes, such as *ACS* and *ACO.* In ethylene’s presence, the receptor senses the ethylene signal and activates EIN2 and EIN3/EIL, activating the ethylene signal transduction cascade, along with signal transmission directed at the ERF family. This directly regulates downstream gene function through specific binding on the downstream gene promoter GCC-box and DRE elements. For example, SlERF.F12 binds to *SlACS2* and *SlACS4* promoters to inhibit their activity, thus inhibiting tomato fruit ripening [12]. MdERF5 is regulated by nitric oxide (NO) and interacts with *MdACO1* at DNA and protein levels, inhibiting its activity and thus fruit ripening [13]. A series of molecular and biochemical experiments suggested the binding between the *MdACS1* promoter and MdERF5L, which negatively regulates its activity, thereby inhibiting fruit ripening. However, the MdWRKY9–MdERF5L interaction weakened the inhibitory effect of MdERF5L on *MdACS1*, which promoted ripening of the fruits. Through EMSA and LUC experiments, we confirmed that MdWRKY9 inhibits *MdERF5L* activity at the transcriptional level, thus inhibiting its function. In other words, MdWRKY9 selectively attaches itself to the W-box on the *MdERF5L* promoter and prevents it from functioning. Also, JrWRKY21 modifies its impact on the downstream gene *JrPR5L* via interacting with JrPTI5L. Furthermore, JrWRKY21 binds to the *JrPTI5L* promoter, suppressing its activity and indirectly controlling the expression of downstream genes that confer resistance to anthrax [18]. We provide a novel ERF transcription factor in the present investigation that is essential for controlling the ripening of fruits. However, whether the interaction between MdWRKY9 and MdERF5l directly or indirectly influence the molecular network of fruit ripening through other signaling pathways or ethylene biosynthesis regulators remains to be fully elucidated. This uncertainty arises from their observed effects on *MdACO1* expression, despite the absence of putative binding sites for either transcription factor in the *MdACO1* promoter. These results explain the regulatory mechanism underlying fruit ripening, and provide novel directions for future research in this field.

MAPK-mediated phosphorylation primarily regulates WRKY transcription factor activity [39]. MPK3/MPK6 phosphorylates AtWRKY33 in *Arabidopsis*, which in turn favorably controls the manufacture of plant antitoxins [40]. Conversely, WRKY34 lacking MPK3/MPK6 phosphorylation sites showed an altered function *in vivo* [41]. Being a negative feedback regulator of MPK3/MPK6, OsWRKY53 plays an inhibitory role in early defense induction [42]. OsMAPKK4-OsMAPK6 phosphorylates and cascades with it to control the biological activity of the BR reaction [43]. Additionally, ICE1 phosphorylation mediated by MPK6 as well as MPK3 lowers its stability, thus negatively regulating plant frost resistance [44, 45]. We here found that MdMAPK6 interacts with MdWRKY9 to stabilize its activity and influence its regulatory role in fruit ripening. MAPK phosphorylation exerts a regulatory influence on fruit ripening and qualitative traits. For instance, phosphorylation of MdNAC72 by MdMAPK3 facilitates the softening of apple fruit [46]. Furthermore, MdMAPK4-mediated phosphorylation of MdERF17 [47] and MYB1 [48] has been shown to promote apple peel degreening and light-induced anthocyanin accumulation, respectively. However, whether MAPK6-mediated phosphorylation of WRKY9 regulates fruit ripening in other climacteric fruits remains to be validated. Furthermore, given the broad-spectrum roles of kinase MAPK and WRKY transcription factors in stress responses [49], it remains unclear whether MAPK6 phosphorylation of WRKY9 mediates the balance between plant stress resistance and fruit quality formation. This work provides novel insights into the regulatory mechanisms of MAPK-mediated post-translational modifications in horticultural crops.

In summary, we elucidated the mechanism through which MdWRKY9 promotes ripening: MdWRKY9 binds to the inhibitor MdERF5L at both protein and DNA levels, thereby inhibiting MdERF5L-mediated regulatory effect on downstream *MdACS1* and promoting fruit ripening. MdWRKY9 also interacts with MdMAPK6; in this interaction, MdMAPK6 phosphorylates and modifies MdWRKY9 at Tyr394 site to stabilize its activity, thus affecting MdWRKY9’s regulatory effect on fruit ripening.

## Materials and methods

### Plant sample and culture conditions

The Guanxian Fruit Tree Breeding Base of Liaocheng City, Shandong Province, China (36°29′N, 115°27′E) provided apples, including offspring lines and the cultivar ‘Taishanzaoxia’. The Bai Shui Apple Experimental Station of Northwest A&F University (35°02′N, 109°06′E) provided the ‘Ruixue’ fruit. For development stage sampling, the first collection was made 45 days after complete bloom, with subsequent collections conducted at a 15-day interval until fruit maturity, resulting in six sampling points. For transient instantaneous injection experiments, near-mature fruits were chosen, injected per experimental procedures, and then kept in a growth chamber at 24°C with a 16-h light and 8-h dark cycle.

Firefly LCI and dual-luciferase tests used *Nicotiana benthamiana* plants, whereas genetic transformation used ‘Micro-Tom’ plants. Both were grown in a plant culture room at 23°C with a relative humidity of 70% ± 5% and a 16-h light and 8-h dark cycle.

### Apple fruits show gene silencing induced by the virus and transient overexpression

The coding arrangements (CDSs) of *MdWRKY9*, *MdWRKY9^Y394D^*, *MdERF5L*, *MdMAPK6*, and *CAMAPK6* (a self-phosphorylating MAPK6 protein formed after a point mutation) [48]. To create recombinant plasmids, the pRI101-GFP overexpression vector was used for cloning. The *A. tumefaciens* GV3101 strain was infected with the mentioned plasmids to generate positive transformants, which were stored at −80°C. The P19 strain and positive bacterial cultures (OD600 = 1.0) were combined in a 1:1 (v:v) ratio. An infection buffer (10 mM MES, 150 μM acetosyringone, and 10 mM MgCl_2_) was used for resuspension and then vacuum-infiltrated into apples. The fruits were completely submerged in the infection solution, and the process was repeated twice under the pressure of 0.8 kg/cm^2^ for 3 min.

Recombinant constructs were created by cloning 400-bp gene-specific CDS segments of *MdERF5L*, *MdMAPK6*, and *MdWRKY9* into the pTRV2 silencing vector. By employing the freeze–thaw technique, these constructs were converted into the *A. tumefaciens* GV3101 strain. The pTRV1 strain was used as a catalyst, and apples were injected with the resulting silencing vectors at the same injection concentration and using the same vacuum infiltration method as for overexpression.

### Heterologous transformation of tomato

Using the freeze–thaw technique, the *MdWRKY9*-pRI101-GFP recombinant vector was added to the *A. tumefaciens* LBA4404 strain. To choose resistant shoots, tomato cotyledons were submerged in an *Agrobacterium* suspension (OD600 = 0.6) for 15 min before being placed on Murashige and Skoog medium that contained 50 mg·l^−1^ kanamycin. A half-strength medium containing 0.1 mg·l^−1^ indole-3-acetic acid was used to root the seedlings. They were transferred to soil to allow their acclimatization and growth, and transgenic seeds were obtained. Transgenic lines were detected through PCR, and subsequent tests used T3 plants.

### Extracting total RNA and RT-qPCR

We used the FastPure® Plant Total RNA Isolation Kit (Vazyme, Nanjing, China) to isolate total RNA. We used the first-strand cDNA Synthesis Kit (TianGen, https://en.tiangen.com) to synthesize cDNA. SYBR Premix Ex Taq II (Takara, Dalian, China) helped in the RT-qPCR. Table S3 lists the used primers. The 2^−ΔΔCT^ technique was used to estimate relative gene expression levels [50]. To guarantee reproducibility, every test was carried out three times.

### Y2H assay

The *MdWRKY9-BD* construct was optimized to remove self-activation based on previously described methods [21]. The pGADT7 vector was used to clone CDSs encoding the interacting proteins *MdERF5L*/*MdMAPK6* to generate the recombinant plasmids *MdERF5L*/*MdMAPK6*-AD. To investigate interactions with *MdWRKY9*-BD, we cotransformed these constructs into yeast cells. Interaction specificity was determined using standard Y2H procedures [3]. β-galactosidase activity was measured using X-α-gal, and blue plaques denoted interactions between proteins. Table S3 lists the primer sequences.

### Pull-down assay

The pET-32a (+) and pGEX-4T-1 vectors were used to clone *MdMAPK6*, *MdERF5L*, and *MdWRKY9* CDSs. To produce GST- and His-tagged fusion proteins, BL21 cells were used to introduce the recombinant plasmids. We used a protein purification kit (Beyotime Biotechnology, Shanghai, China) to purify them. To establish an effective association, the eluted proteins were subjected to western blotting using anti-His and anti-GST antibodies (Abmart, Shanghai, China).

### BiFC assay

Whereas the pSPYCE-YFP vector cloned the *MdMAPK6* and *MdERF5L* CDSs, the pSPYNE-YFP vector cloned the *MdWRKY9* CDS. We used sterile syringes to convert the recombinant constructs into competent cells of the *A. tumefaciens* GV3101 strain, followed by infiltration into *N. benthamiana* leaves. A confocal microscope was used to view the YFP signals following 2 days of infiltration. Direct interactions between proteins were revealed by fluorescence.

### Firefly LCI assay

The pCAMBIA1300-LUC^N^ vector cloned the *MdMAPK6* and *MdERF5L* full-length CDSs, whereas the pCAMBIA1300-LUC^C^ vector cloned that of *MdWRKY9*. The *A. tumefaciens* strain GV3101 was used to transform recombinant plasmids. The goal was to co-infiltrate agrobacterial suspensions into 5-week-old *N. benthamiana* leaves. A total of 2.5 days following inoculation, we photographed luminescence signals. Table S3 lists the primers.

### EMSA

Similar to the pull-down assay, we transformed *Escherichia coli* BL21 cells and induced them to produce purified proteins, which were subsequently stored at −80°C for further analysis. Using PlantCARE software, we analyzed *cis*-acting elements within *MdERF5L* and *MdACS1* promoters to determine potential binding sites. To evaluate interactions between proteins and DNA, unlabeled competitive, mutant, and biotin-labeled probes were developed. The LightShift Chemiluminescent EMSA kit (Thermo) was used for EMSAs per the manufacturer’s protocol. Table S3 lists the probe sequences.

### LUC reporter test

We used the pGreenII 62-SK vector to clone the CDSs of *MdERF5L* and *MdWRKY9*, and the pGreenII 0800-LUC vector to clone *MdACS1* and *MdERF5L* promoters. The mentioned recombinant constructs were added to *A. tumefaciens* LBA4404 cells, together with the P19 helper plasmid. Tobacco leaves were co-infected according to experimental requirements, and an *in vivo* imaging system captured each promoter’s fluorescein intensity. The dual-luciferase reporter gene assay kit [Yeasen Biotechnology (Shanghai) Co., Ltd.] detected luciferase activity. Table S3 contains a list of all primers.

### ChIP-PCR examination

We conducted the ChIP-PCR examination with the *MdWRKY9*-GFP calli that were acquired. Primers were created to target the *MdERF5L* promoter’s W-box region (~200 bp) [3]. PCR was used to amplify the immunoprecipitated DNA fragments. Table S3 contains a list of all primer sequences.

### GUS activity test

The *proMdERF5L*::GUS vector was created by inserting the *MdERF5L* promoter into the pCAMBIA1305-GUS vector. The *Agrobacterium*-mediated transformation technique was used to produce the positive transgenic calli. Using empty promoter (Empty::GUS) as negative control, the 35S::MdWRKY9 and 35S::CAMdMAPK6 were transiently transformed into *proMdERF5L*:GUS calli. After co-culture for 2 days, the calli were subjected to GUS staining and assayed for activity.

### LC–MS/MS analysis and *in vitro* phosphorylation

The IP/MS analysis was conducted by Beijing Biotech Packaging Technology Co., Ltd. to identify phosphorylation at the Tyr394 residue of *MdWRKY9*. To mimic phosphorylation, the Tyr394 residue was replaced with aspartic acid (*MdWRKY9^T394D^*). The pGEX4T-1-GST vector was used to clone full-length *MdWRKY9* and *MdWRKY9^Y394D^* CDSs. The pET-32a (+) vector was used to clone *CAMdMAPK6* CDS, encoding the active kinase. *Escherichia coli* BL21 was used to express recombinant proteins; they were purified and subjected to *in vitro* kinase assays, as described elsewhere [51]. Briefly, the induced proteins were mixed in a 1:5 ratio according to the target and underwent incubation for 40 min at 30°C in a kinase reaction buffer. The phosphorylation of MdMAPK6 on MdWRKY9 was detected through western blotting by using GST, His, and phosphorylation (PhophoS/T/Y) antibodies. Table S3 contains a list of the primers.

### Cell-free protein degradation experiment

Both *MdMPK6* silencing (-TRV2) and overexpression (-OE) and WT calli had their crude proteins removed. We added recombinant MdWRKY9-GST to the total protein extract and incubated it for the specified amount of time. They were immunoblotted using an anti-GST antibody, with WT serving as the control. In addition, purified His-tagged proteins were used as controls. CAMdMPK6-His was reacted with MdWRKY9-GST and MdWRKY9^Y394D^-GST in an ATP-containing kinase reaction buffer for 0, 20, 40, and 60 min. We conducted western blotting to evaluate MdWRKY9’s degradation rate.

### Texture assessment

Fruit hardness was assessed using a CTX texture analyzer (AMETEK Brookfield, Middleborough, MA, USA) with the following parameters: a 2 mm probe diameter, 10 mm penetration depth, 1 mm/s descending speed, 1 mm/s penetration speed, and 10 mm/s measurement speed. For every fruit, measurements were made three times at three different equatorial positions.

### Determination of ethylene release levels

The Clarus 580 GC system (PerkinElmer, Waltham, MA, USA) equipped with a Porapak Q column (80/100 mesh, 0.32 mm internal diameter) for quantification of ethylene levels. The column oven was maintained at 200°C in isothermal mode. Fruit samples were sealed in 1-l headspace vials and equilibrated at 25°C for 24 h to allow ethylene accumulation. A 1-ml headspace gas sample was manually injected into the GC system. Ethylene concentrations were calculated based on peak area integration and expressed as release rate per unit time per unit mass of fruit. Each sample was assessed at least three times.

### Statistical tests

A minimum of three technical and three biological replicates were used in every experiment. The mean ± standard deviation (SD) of three separate biological replicates is used to show the data. The Student’s *t* test with SPSS Statistics 22 (IBM Corporation, Armonk, NY, USA) was used to establish statistical significance (*P* < 0.05 and *P* < 0.01). The SD of the replicates is shown by error bars in the figures. Microsoft Excel was used to process the data.

## Supplementary Material

Web_Material_uhaf200

## Data Availability

All supporting data are included in this paper or the supplementary materials.
