# Supplementary material for: MdMAPK6-mediated phosphorylation of MdWRKY9 regulates apple fruit ripening through interaction with MdERF5L
Source: Hortic Res. 2025 Jul 31;12(11):uhaf200. doi: 10.1093/hr/uhaf200 (PMC12554370; doi:10.1093/hr/uhaf200)
Supplement: Web_Material_uhaf200 [file web_material_uhaf200.zip › Supplemental Figure S.docx]

**Supplemental Figure S1.** Semi-quantitative analysis of *MdWRKY9* and expression level analysis of key ethylene synthesis genes in ‘Taishanzaoxia’ apples transiently transformed with MdWRKY9. **(A)** The semi-quantitative analysis results of MdWRKY9. (B-C) Analysis results of the expression levels of *MdACS1* **(B)** and *MdACO1* (C) genes. The SD of three separate biological replicates is shown by error bars. Significant differences at *P* < 0.05 are indicated via various lowercase letters (Student’s *t* test).


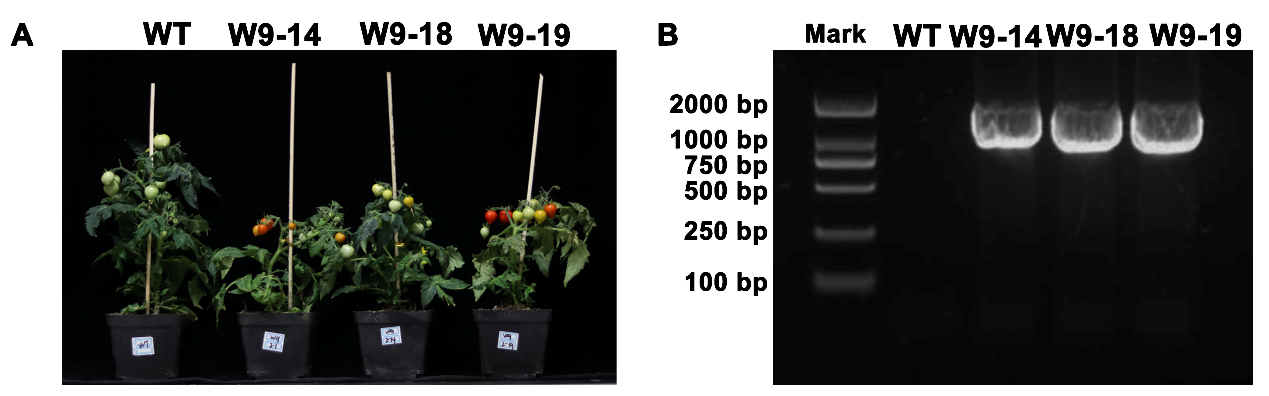


**Supplemental Figure S2.** The phenotype **(A)** and PCR positive test results **(B)** of *MdWRKY9* transgenic tomato lines.


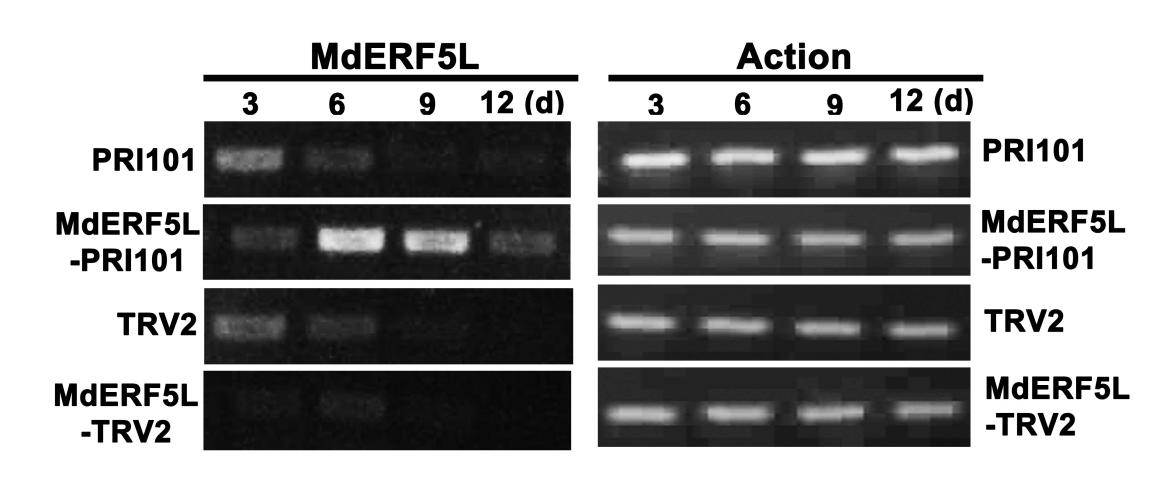


**Supplemental Figure** **S3.** Semi-quantitative results of transient injection of *MdERF5L* in ‘Taishanzaoxia’ apple.


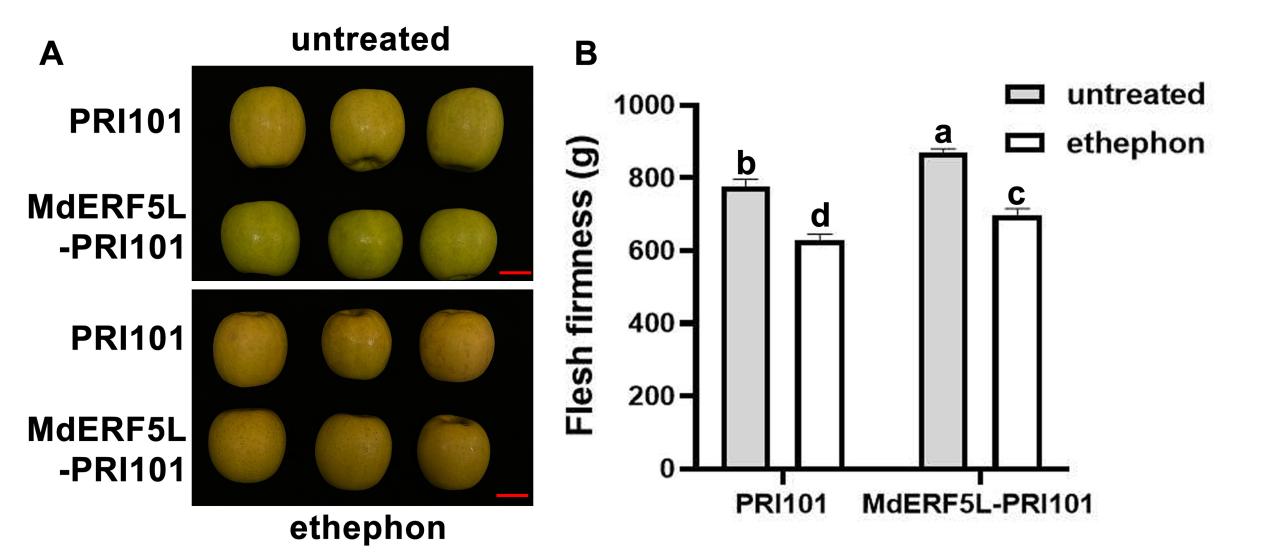


**Supplemental Figure** **S4.** Effects of ethephon treatment and overexpression of *MdERF5L* on the ripening of ‘Ruixue’ fruits. Phenotype **(A)** and flesh firmness **(B)** after 9 days of treatment. Bar = 4 cm. The treatment concentration of ethephon was 1,000 mg·L^−1^ for 1 minute. Error bars represent the SD of three independent biological replicates. Different lowercase letters indicate significant differences at *P <* 0.05 (Student’s *t* test).


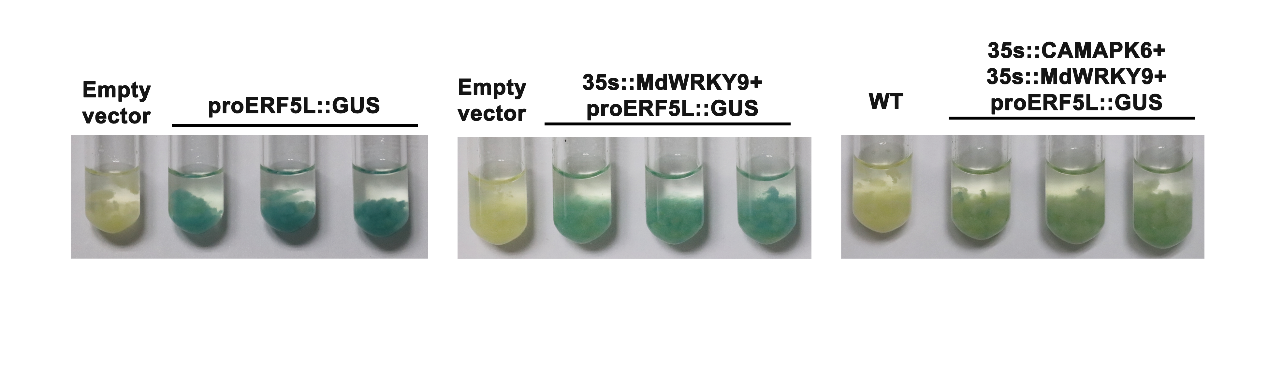


**Supplemental Figure** **S5.** The GUS staining phenotype of *ProERF5L*::GUS calli with transient expression of *MdWRKY9* and *MdMAPK6*.
