# Supplementary material for: MdMAPK6-mediated phosphorylation of MdWRKY9 regulates apple fruit ripening through interaction with MdERF5L
Source: Hortic Res. 2025 Jul 31;12(11):uhaf200. doi: 10.1093/hr/uhaf200 (PMC12554370; doi:10.1093/hr/uhaf200)
Supplement: Web_Material_uhaf200 [file web_material_uhaf200.zip › Supplemental Table S1.docx]

**Table S1 The proteins identified in a MdWRKY9 yeast two-hybrid library interaction.**

| Gene ID | Annotated Function |
| --- | --- |
| MD10G1314900 | Ubiquitin [*Medicago* *truncatula*] |
| MD01G1139500 | BTB/POZ and MATH domain-containing protein 3-like isoform X1 [*Pyrus* *bretschneideri*] |
| MD14G1215200 | alpha-1,4-glucan-protein synthase [UDP-forming] [*Malus* *domestica*] |
| MD04G1030300 | chitinase-like protein 1 [*Malus* *domestica*] |
| MD12G1057600 | omega-6 fatty acid desaturase, endoplasmic reticulum isozyme 2 [*Malus* *domestica*] |
| MD06G1051900 | ethylene responsive element binding factor 5 |
| MD06G1124100 | flavonol synthase/flavanone 3-hydroxylase-like [*Malus* *domestica*] |
| MD17G1034800 | formate dehydrogenase, mitochondrial [*Malus* *domestica*] |
| MD09G1178600 | protein TIFY 11B-like [*Malus* *domestica*] |
| MD04G1044600 | 60S ribosomal protein L3 [*Malus* *domestica*] |
| MD06G1114800 | serine/threonine-protein kinase WNK8-like [*Malus* *domestica*] |
| MD03G1211300 | polyubiquitin 10 [*Arabidopsis* *thaliana*] |
| MD05G1341400 | polyubiquitin 10 [*Arabidopsis* *thaliana*] |
| MD17G1164400 | protein TIFY 10B-like [*Malus* *domestica*] |
| MD00G1160700 | LOW QUALITY PROTEIN: guanine nucleotide-binding protein subunit beta-like protein [*Malus* *domestica*] |
| MD01G1148400 | probable aquaporin PIP1-4 [*Malus* *domestica*] |
| MD07G1009000 | ADP,ATP carrier protein 1, mitochondrial-like [*Malus* *domestica*] |
| MD02G1072100 | vacuolar protein sorting-associated protein 32 homolog 2-like [*Malus* *domestica*] |
| MD15G1217900 | thiol protease aleurain-like [*Malus* *domestica*] |
| MD15G1073300 | 3-oxo-Delta(4,5)-steroid 5-beta-reductase-like [*Malus* *domestica*] |
| MD02G1004000 | MAP protein kinase 6 |
| MD17G1001600 | carbonyl reductase family member 4-like [*Malus* *domestica*] |
| MD04G1127400 | actin [*Malus* *domestica*] |
| MD07G1174700 | probable aquaporin PIP2-5 [*Malus* *domestica*] |
| MD17G1066000 | vegetative cell wall protein gp1-like [*Malus* *domestica*] |
| MD13G1185100 | stem-specific protein TSJT1 [*Pyrus* *bretschneideri*] |
| MD13G1060200 | probable 6-phosphogluconolactonase 2 [*Malus* *domestica*] |
| MD11G1241700 | protochlorophyllide reductase, chloroplastic-like [*Malus* *domestica*] |
