# Supplementary material for: MdMAPK6-mediated phosphorylation of MdWRKY9 regulates apple fruit ripening through interaction with MdERF5L
Source: Hortic Res. 2025 Jul 31;12(11):uhaf200. doi: 10.1093/hr/uhaf200 (PMC12554370; doi:10.1093/hr/uhaf200)
Supplement: Web_Material_uhaf200 [file web_material_uhaf200.zip › Supplemental Table S2.docx]

**Table S2 Identification of phosphorylated peptides based on LC-MS/MS.**

| Peptide  <ProteinMetricsConfidential > | Observed  m/z | z | Mass error  (ppm) | Score | Scan Time | Intensity |
| --- | --- | --- | --- | --- | --- | --- |
| R.KY[+79.966]GQKVVK.G，Position-394（Y） | 515.2881 | 2 | 19.2 | 393 | 6.7889 | 20453000 |
| R.PLALPNHSNLR.Y | 616.3376 | 2 | -18.4 | 474 | 28.4021 | 795140000 |
| R.KHVER.A | 668.397 | 1 | 19.7 | 420 | 21.573 | 13286000 |
| R.FTDRNGTEIPKFR.S | 790.9251 | 2 | 15.8 | 39 | 21.0267 | 9311400 |
| K.DTQQGIRESEPK.M | 694.3499 | 2 | 8.1 | 116 | 25.6672 | 10802000 |

Note: The information in the samples collected by LC-MS/MS mass spectrometry was retrieved by Byonic database, and the phosphorylpeptide information in MdWRKY9-GFP protein was identified. Peptide represents the amino acid sequence identified to the peptide segment; m/z represents the mass-charge ratio of the peptide; Score represents for peptide score; Scan represents the mass spectrometry number for which the peptide has been identified; Intensity represents the abundance of the peptide; S,T,Y[+79.966]: Phospho (STY) phosphorylation occurs on serine, threonine and tyrosine with molecular weight of +79.966 Da.
