# Supplementary material for: MdMAPK6-mediated phosphorylation of MdWRKY9 regulates apple fruit ripening through interaction with MdERF5L
Source: Hortic Res. 2025 Jul 31;12(11):uhaf200. doi: 10.1093/hr/uhaf200 (PMC12554370; doi:10.1093/hr/uhaf200)
Supplement: Web_Material_uhaf200 [file web_material_uhaf200.zip › Supplemental Table S3.docx]

**Table S3 Primers used in this study.**

| **Role** | **Name** | **primer (5′-3′)** |
| --- | --- | --- |
| **transgene** | **MdWRKY9(PRI101-GFP)-F** | **ttgatacatatgcccgtcgacATGACTTCTTCCTTCACGAACCTC** |
|  | **MdWRKY9(PRI101-GFP)-R** | **tcagaattcggtacccccgggGAACTCAGAAAATCCATAACTTCCTTC** |
|  | **MdERF5L(PRI101-GFP)-F** | **ttgatacatatgcccgtcgacATGGCTTTAGATCAGGTCTCGG** |
|  | **MdERF5L(PRI101-GFP)-R** | **tcagaattcggtacccccgggTACGACCATAAGCTGTGGACAACC** |
|  | ***MdMAPK6*(PRI101-GFP)-F** | **ttgatacatatgcccgtcgacATGGAGGGAGGAGGGCGA** |
|  | ***MdMAPK6*(PRI101-GFP)-R** | **tcagaattcggtacccccgggCTGTCGCTGGTACTCGGGG** |
|  | **ProERF5L(1305-GUS)-F** | **tatgaccatgattacgaattcAGGAGTTATGACAATTTTTTGGACC** |
|  | **ProERF5L(1305-GUS)-R** | **caggtcgactctagaggatccTGATAACAATTTTTTTAGCAAGTATCTATTT** |
|  | **TRV2-MdWRKY9-F** | **gtgagtaaggttaccgaattcCAGTCTACAAGAAGATCAAGCTCGA** |
|  | **TRV2-MdWRKY9-R** | **gagacgcgtgagctcggtaccATGTGCATTTGTAGTAGCTCCTCG** |
|  | **TRV2-MdMAPK6-R** | **gagacgcgtgagctcggtaccAGGAAGCTGCCGAATGTATCTC** |
|  | **TRV2-MdERF5L-F** | **gtgagtaaggttaccgaattcTGGCTTTAGATCAGGTCTCGGC** |
|  | **TRV2-MdERF5L-F** | **gtgagtaaggttaccgaattcTGGCTTTAGATCAGGTCTCGGC** |
|  | **TRV2-MdERF5L-R** | **gagacgcgtgagctcggtaccGCTTTTGCTCAAATTCAAAGTAATCG** |
| **Gene expression** | **MdWRKY9-F** | **CCGATAATAGCAGCAACAAT** |
|  | **MdWRKY9-R** | **TCCATAACTTCCTTCACTCT** |
|  | **MdERF5L-F** | **GTCAGGAACACAGAGAAGA** |
|  | **MdERF5L-R** | **GAGATAACGGCGACAATG** |
|  | **MdACS1-F** | **ACTTGCTTAGGTCCAACA** |
|  | **MdACS1-R** | **GAGATATTGAGGCGAACTTC** |
|  | **MdACO1-F** | **TCTTTGAGCTGGTGAACC** |
|  | **MdACO1-R** | **TCCTTAAACCTTTGCTCCAT** |
|  | **MdActin-F** | **TGACCGAATGAGCAAGGAAATTACT** |
|  | **MdActin-F** | **TACTCAGCTTTGGCAATCCACATC** |
| **Y2H** | **MdWRKY9(pGBKT7)-F** | **tcagaggaggacctgcatatgATGTCTACTCAATCTTCGCAATATGG** |
|  | **MdWRKY9(pGBKT7)-R** | **ccgctgcaggtcgacggatccGAACTCAGAAAATCCATAACTTCCTTC** |
|  | **MdERF5L(pGADT7)-F** | **gtaccagattacgctcatatgATGGCTTTAGATCAGGTCTCGG** |
|  | **MdERF5L(pGADT7)-R** | **cagctcgagctcgatggatccTACGACCATAAGCTGTGGACAACC** |
|  | **MdMAPK6(pGADT7)-F** | **gtaccagattacgctcatatgATGGAGGGAGGAGGGCGA** |
|  | **MdMAPK6(pGADT7)-R** | **cagctcgagctcgatggatccCTGTCGCTGGTACTCGGGG** |
| **BIFC** | **MdWRKY9(pUC-SPYCE)-F** | **ggatccATGACTTCTTCCTTCACGAACCTC** |
|  | **MdWRKY9(pUC-SPYNE)-R** | **gtcgacGAACTCAGAAAATCCATAACTTCCTTC** |
|  | **MdERF5L(pUC-SPYNE)-F** | **ggatccATGGCTTTAGATCAGGTCTCGG** |
|  | **MdERF5L(pUC-SPYCE)-R** | **gtcgacTACGACCATAAGCTGTGGACAACC** |
|  | **MdMAPK6(pUC-SPYCE)-F** | **ggatccATGGAGGGAGGAGGGCGA** |
|  | **MdMAPK6(pUC-SPYCE)-R** | **ggatccCTGTCGCTGGTACTCGGGG** |
| **Inducible protein** | **MdWRKY9(PET32a)-F** | **gccatggctgatatcggatccATGACTTCTTCCTTCACGAACCTC** |
|  | **MdWRKY9(PET32a)-R** | **ttgtcgacggagctcgaattcGAACTCAGAAAATCCATAACTTCCTTC** |
|  | **MdWRKY9(PGEX4T-1)-F** | **gatctggttccgcgtggatccATGACTTCTTCCTTCACGAACCTC** |
|  | **MdWRKY9(PGEX4T-1)-R** | **ctcgagtcgacccgggaattcGAACTCAGAAAATCCATAACTTCCTTC** |
|  | **MdERF5L(PGEX4T-1)-F** | **gatctggttccgcgtggatccATGGCTTTAGATCAGGTCTCGG** |
|  | **MdERF5L(PGEX4T-1)-R** | **ctcgagtcgacccgggaattcTACGACCATAAGCTGTGGACAACC** |
|  | **MdMAPK6(PGEX4T-1)-F** | **ctcgagtcgacccgggaattcATGGAGGGAGGAGGGCGA** |
|  | **MdMAPK6(PGEX4T-1)-R** | **ctcgagtcgacccgggaattcCTGTCGCTGGTACTCGGGG** |
|  | **MdMAPK6(PET32a)-F** | **gccatggctgatatcggatccATGGAGGGAGGAGGGCGA** |
|  | **MdMAPK6(PET32a)-R** | **ttgtcgacggagctcgaattccCTGTCGCTGGTACTCGGGG** |
| **LCI assay** | **MdWRKY9(cLUC)-F** | **TCCCGGGGCGGTACCATGACTTCTTCCTTCACGAACCTC** |
|  | **MdWRKY9(cLUC)-R** | **CTGCAGGTCGACATGACTTCTTCCTTCACGAACCTC** |
|  | **MdERF5L(nLUC)-F** | **GAGCTCGGTACCCGGGATCCATGGCTTTAGATCAGGTCTCGG** |
|  | **MdERF5L(nLUC)-R** | **CGAGATCTGGTCGACTACGACCATAAGCTGTGGACAACC** |
|  | **MdMAPK6(nLUC)-F** | **GAGCTCGGTACCCGGGATCCATGGAGGGAGGAGGGCGA** |
|  | **MdMAPK6(nLUC)-R** | **CGAGATCTGGTCGACCTGTCGCTGGTACTCGGGG** |
| **EMSA** | **MdACS1-DRE(hot probe)-F** | **AAAATAGAGGAGAAAAATGGCCGACAAAATTTGTTGTGGCTACTA** |
|  | **MdACS1-DRE(hot probe)-R** | **TAGTAGCCACAACAAATTTTGTCGGCCATTTTTCTCCTCTATTTT** |
|  | **MdACS1-DRE(cold probe)-F** | **AAAATAGAGGAGAAAAATGGCCGACAAAATTTGTTGTGGCTACTA** |
|  | **MdACS1-DRE(cold probe)-R** | **TAGTAGCCACAACAAATTTTGTCGGCCATTTTTCTCCTCTATTTT** |
|  | **MdACS1-DRE(mutant probe)-F** | **AAAATAGAGGAGAAAAATGGTCTAGAAAATTTGTTGTGGCTACTA** |
|  | **MdACS1-DRE(mutant probe)-R** | **TAGTAGCCACAACAAATTTTCTAGACCATTTTTCTCCTCTATTTT** |
|  | **MdERF5L-W-box(hot probe)-F** | **AATTTGTATATCGTTTTCTGGGTCAATGATACGTTTTTTTATATTA** |
|  | **MdERF5L-W-box(hot probe)-R** | **TAATATAAAAAAACGTATCATTGACCCAGAAAACGATATACAAATT** |
|  | **MdERF5L-W-box(cold probe)-F** | **AATTTGTATATCGTTTTCTGGGTCAATGATACGTTTTTTTATATT** |
|  | **MdERF5L-W-box(cold probe)-R** | **TAATATAAAAAAACGTATCATTGACCCAGAAAACGATATACAAATT** |
|  | **MdERF5L-W-box(mutant probe)-F** | **AATTTGTATATCGTTTTCTGGATTAGTGATACGTTTTTTTATATTA** |
|  | **MdERF5L-W-box(mutant probe)-R** | **TAATATAAAAAAACGTATCACTAATCCAGAAAACGATATACAAATT** |
| **LUC** | **MdWRKY9(pGreenⅡ62-SK)-F** | **cgctctagaactagtggatccATGACTTCTTCCTTCACGAACCTC** |
|  | **MdWRKY9(pGreenⅡ62-SK)-R** | **gtcgacggtatcgataagcttGAACTCAGAAAATCCATAACTTCCTTC** |
|  | **MdERF5L(pGreenⅡ62-SK)-F** | **cgctctagaactagtggatccATGGCTTTAGATCAGGTCTCGG** |
|  | **MdERF5L(pGreenⅡ62-SK)-R** | **gtcgacggtatcgataagcttATGGCTTTAGATCAGGTCTCGG** |
|  | **proMdACS1(pGreenⅡ0800-Luc)-F** | **gggccccccctcgaggtcgacGCTTTCTTTATGCTAATTGCTGAAA** |
|  | **proMdACS1(pGreenⅡ0800-Luc)-R** | **cgctctagaactagtggatccTTTGGTAACAAGCTATAACACAAAAGTG** |
|  | **proMdERF5L(pGreenⅡ0800-Luc)-F** | **gggccccccctcgaggtcgacAGGAGTTATGACAATTTTTTGGACC** |
|  | **proMdERF5L(pGreenⅡ0800-Luc)-R** | **cgctctagaactagtggatccTGATAACAATTTTTTTAGCAAGTATCTATTT** |
| **CHIP-PCR** | **proMdERF5L(Contains W-box site)-F** | **GTATATGATATGATAATCGGTAGG** |
|  | **proMdERF5L(Contains W-box site)-R** | **GTCTTGTCTAAGTAATCAAAGTTC** |
| **Point mutation** | **MdMAPK6-F** | **ATGGAGGGAGGAGGGCGA** |
|  | **MdMAPK6-R** | **CTGTCGCTGGTACTCGGGG** |
|  | **CAMdMAPK6-F** | **CCTCAGAGACTGGTTTTATGACTGCGTAT** |
|  | **CAMdMAPK6-R** | **ATACGCAGTCATAAAACCAGTCTCTGAGG** |
|  | **MdWRKY9-F** | **ATGACTTCTTCCTTCACGAACCTCCTC** |
|  | **MdWRKY9-R** | **GAACTCAGAAAATCCATAACTTCC** |
|  | **TBMdWRKY9^Y394D^-F** | **GTGGAGGAAGGATGGACAGAAAG** |
|  | **TBMdWRKY9^Y394D^-R** | **CTTTCTGTCCATCCTTCCTCCAC** |
